# Dynein, microtubule and cargo: a ménage à trois

**DOI:** 10.1042/BST20130235

**Published:** 2013-11-20

**Authors:** Nenad Pavin, Iva M. Tolić-Nørrelykke

**Affiliations:** *Department of Physics, Faculty of Science, University of Zagreb, 10002 Zagreb, Croatia; †Max Planck Institute of Molecular Cell Biology and Genetics, Pfotenhauerstrasse 108, D-01307 Dresden, Germany

**Keywords:** cargo, cortical anchor, dynein, microtubule, motor protein, targeting

## Abstract

To exert forces, motor proteins bind with one end to cytoskeletal filaments, such as microtubules and actin, and with the other end to the cell cortex, a vesicle or another motor. A general question is how motors search for sites in the cell where both motor ends can bind to their respective binding partners. In the present review, we focus on cytoplasmic dynein, which is required for a myriad of cellular functions in interphase, mitosis and meiosis, ranging from transport of organelles and functioning of the mitotic spindle to chromosome movements in meiotic prophase. We discuss how dynein targets sites where it can exert a pulling force on the microtubule to transport cargo inside the cell.

## How motors generate large forces in the cell

The interior of a living cell resembles a tiny city, which requires constant and extensive transport of material between different regions. Transport inside the cell requires forces to move and position various molecular assemblies and organelles in response to progression through the cell cycle and signals from the environment. These forces are mostly generated by motor proteins such as myosin, kinesin and dynein. Myosin moves along actin filaments, whereas kinesin and dynein move along microtubules (reviewed in [1–3]).

**Figure 1:**
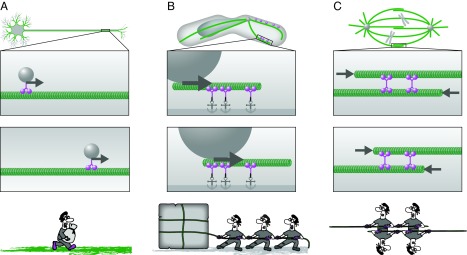
Three scenarios of how motor proteins exert force on cytoskeleton filaments In (**A**)–(**C**), the upper scheme shows the biological context, the two windows in the middle represent two time points to visualize the movement, and the drawings below illustrate a macroscopic equivalent of the process. (**A**) Motors (magenta) use the filaments (green) as tracks to transport cargo (grey sphere), e.g. vesicles in the axon. (**B**) Motors use the filaments as ropes to pull on large cargo, e.g. the nucleus. Here, motors are anchored (anchor signs) at the cell cortex (grey stripe). (**C**) Motors exert pushing force between adjacent cytoskeleton filaments, e.g. in the mitotic spindle. Arrows indicate the direction of force.

The ways in which motor proteins exert force on cytoskeleton filaments can be visualized in three scenarios. First, motors use the filaments as tracks to transport cargo such as organelles, vesicles, proteins and RNA. This can be imagined as carrying load along a road (Figure 1A). This scenario is typical for small cargos, whose transport inside the cell does not require large force. Secondly, motors use the filaments as ropes to pull on structures such as the mitotic spindle, centrosome and the nucleus, which is similar to pulling on a rope in the macroscopic world (Figure 1B). The cell employs this scenario to transport large objects, which require large force to be transported. To pull on them, motors are typically anchored at the cell cortex and exert force against the cortex by walking along the filaments. This configuration allows for accumulation of a higher number of motors and thus generation of larger forces than in the first scenario. In the third scenario, motors together with other cytoskeleton cross-linking proteins exert pushing or pulling force between adjacent cytoskeleton filaments to organize them into higher-order structures such as the mitotic spindle and the cell cortex [4] (Figure 1C). The variety of biological structures and functions in these scenarios result from self-organization of motor proteins and cytoskeleton filaments, but how this process occurs largely remains a mystery.

## Dynein, a motor that exerts force on the microtubules

In the present review, we focus on dynein, which is a motor protein that walks along the microtubule towards the minus end, i.e. the end typically found at the centrosome and exhibiting lower dynamics than the opposite plus end. Dynein transports vesicles, proteins and RNA towards the cell centre [5] and positions the centrosome in interphase [6,7], which has been reconstituted *in vitro* [8,9]. During mitosis, dynein has numerous functions. It localizes at the cell cortex, where it pulls on astral microtubules to position the spindle [10–15]. Moreover, dynein is found at kinetochores, where it regulates microtubule attachment, tension at the kinetochores, chromosome movement and silencing of the spindle assembly checkpoint [16,17]. Dynein also has a role in meiotic prophase, when it generates chromosome movements that are important for pairing of homologous chromosomes [18,19].

A beautiful experimental model systems to study large-scale movements driven by dyneins is the fission yeast *Schizosaccharomyces pombe*, where dynein moves the nucleus back and forth from one end of the cell to the other [18,20]. To drive these oscillations of the nucleus, dynein is anchored at the cell cortex and pulls on astral microtubules [21,22], thus this system belongs to the rope-pulling scenario from Figure 1(B). The force generated by dynein is transmitted to the chromosomes via the microtubules, which are attached to the nucleus via the SUN/KASH domain proteins that span the nuclear envelope and bind to chromosome ends [23,24]. These movements of the chromosomes promote chromosome pairing, recombination and spore viability [18].

## Dynein targets microtubules and the cortex in two steps

**Figure 2:**
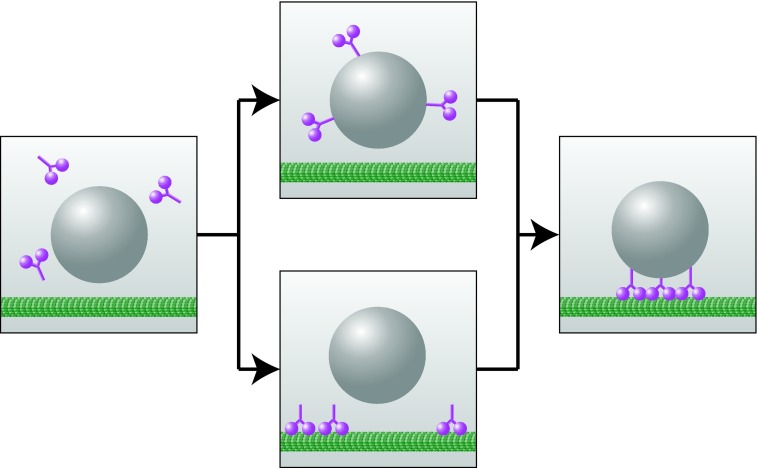
Two targeting mechanisms of dynein Dynein (magenta) from the cytoplasm (grey background) binds first either to the cargo (grey sphere) or to the microtubule (green), as shown by the upper and the lower pathway respectively. Subsequently, dynein also binds the other binding partner, and starts to perform its function in the cell.

To exert forces, dynein binds to two partners: with the head domain to the microtubule and with the tail domain to the cell cortex or cargo. To understand how dynein performs its function in the cell, one needs to consider how dynein targets sites where it can bind simultaneously to a microtubule and to the cargo (Figure 2). This process can occur in two ways: dynein from the cytoplasm binds first either to the microtubule or to the cargo, and subsequently to the other binding partner.

A direct way to experimentally identify these steps is to observe individual dynein molecules in a living cell [25]. We have recently managed to follow single dyneins during nuclear oscillations in fission yeast, by using a TIRF (total internal reflection fluorescence) microscopy set-up [26]. In this system, dynein binds to the microtubule and to the cortex, rather than to cargo, in order to pull on the microtubule. In our single-molecule experiments, we were able to track individual dynein molecules diffusing in the cytoplasm. These experiments allowed us to directly visualize binding of dynein from the cytoplasm to the microtubule and from the microtubule to the cortex, and thus to identify the sequence of steps of the targeting mechanism (Figure 2, lower pathway).

This targeting mechanism may be conserved in other organisms. For example, the last step of the targeting mechanism, where dynein from the microtubule binds to the cortex, which is termed ‘off-loading’, was identified in budding yeast [27–30]. In this system, dynein anchored at the cortex pulls on the microtubules in order to move the mitotic spindle into the bud. It will be interesting to observe all steps of dynein binding and thus to complete the picture of the targeting mechanism in budding yeast.

Binding of dynein from the cytoplasm first to the microtubule, as opposed to first to the cortex, may be beneficial for the cell. If dyneins bound to the cortex first, most of them would remain at those parts of the cortex that would not be visited by microtubules. In contrast, if dyneins bind to the microtubule first, it brings dynein to the cortex because most microtubules reach the cortex. The situation may be different when dynein carries small cargo along the microtubules instead of pulling on the microtubule against the cortex. Here, it may be advantageous for dynein to bind first to the cargo (Figure 2, upper pathway), and future experiments will reveal the targeting mechanism in this case.

## Nuclear oscillations rely on the asymmetric pattern of dynein

Diffusion through the cytoplasm allows dyneins to explore the intracellular space and target microtubules at different locations in the cell. In other words, by moving in the cytoplasm, dyneins redistribute throughout the cell in order to self-organize into a pattern required to generate nuclear oscillations. During the oscillations, dyneins detach from the cortex in response to higher load forces, which occurs on the microtubule trailing behind the nucleus. At the same time, dyneins accumulate to sites where they experience lower load forces, which happens on the microtubule leading the nucleus. Thus, during nuclear movement, there will be more dyneins on the microtubule in front of the nucleus than behind it, even when these two microtubules are equally long as the nucleus is passing through the cell centre. In other words, there is information in the system about the direction of movement, which is used as memory to maintain the same direction as the nucleus is moving from one end of the cell to the other, thereby generating regular oscillatory movement [31,32].

## Movement of dynein along the microtubule: directed or diffusive?

**Figure 3:**
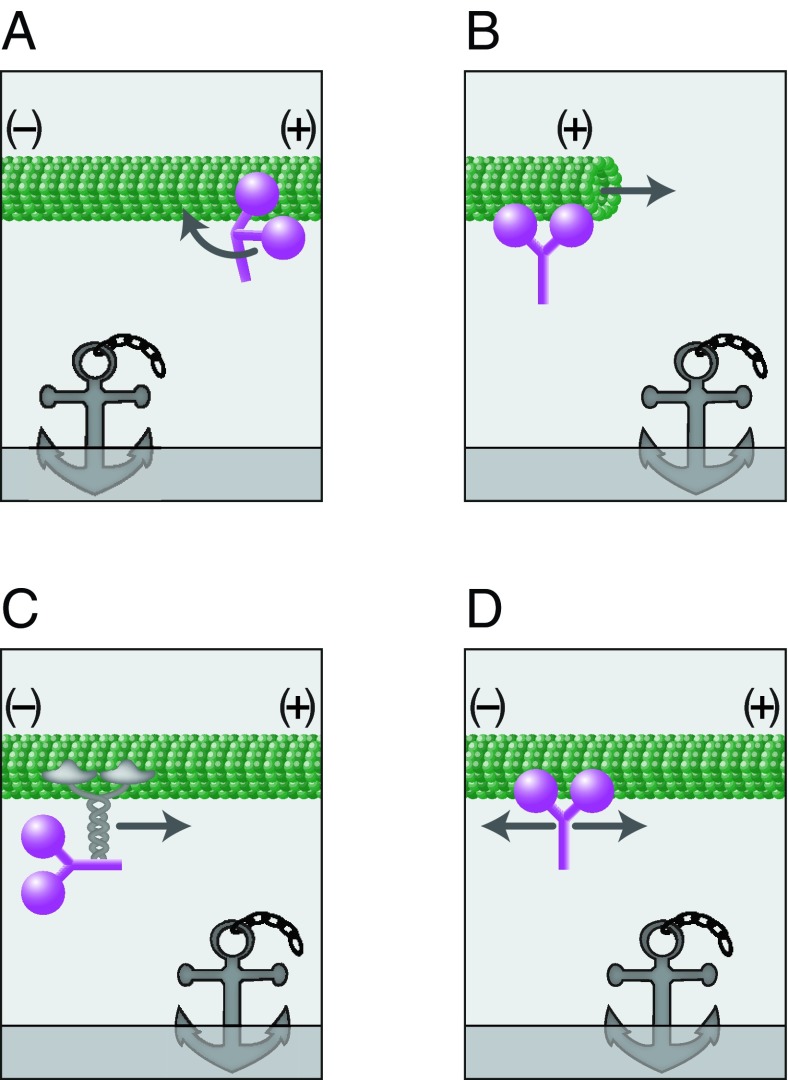
Four types of dynein movement on the microtubule On the microtubule (green), dynein (magenta) may: (**A**) move towards the minus end, (**B**) follow the growing plus end, (**C**) be transported towards the plus end by a kinesin (grey motor), or (**D**) diffuse along the microtubule. By moving along the microtubule, dynein eventually finds an anchor protein (anchor sign) at the cortex.

To understand the biological roles of dynein, it is important to know how dynein behaves upon binding to the microtubule. In principle, dynein may move towards either the minus or the plus end, or not move in a directed manner (Figure 3). Experiments in different systems *in vivo* have shown different dynein behaviour. In budding yeast, dynein follows the plus end of the growing microtubule in a Bik1/CLIP (cytoplasmic linker protein)-170- and Pac1/LIS1-dependent manner, as a result of transport by the kinesin Kip2 along the microtubule in the direction of the plus end, or as a consequence of direct binding from the cytoplasm to the plus end [33,34]. When the plus end brings dynein close to the cortical anchors, dynein binds to the anchors in a process known as off-loading [28]. In the plant pathogenic fungus *Ustilago maydis*, kinesin-1 transports dynein towards the plus end of the microtubules within the tip of the infectious hypha [35,36]. In HeLa cells, dynein has been observed to form two types of foci: spot-like, which move either towards the plus or the minus end of the microtubule, and comet-like foci, which follow the plus ends of the growing microtubules [37]. In contrast with these systems, dynein in fission yeast does not move towards either end of the microtubule, but instead performs one-dimensional diffusion along the microtubule upon binding from the cytoplasm [26].

Diffusion of dynein along the microtubule may allow dynein to search for target sites in the neighbourhood. Because diffusion is efficient for exploring space on a short-length scale, dynein may use diffusion as a search strategy for target sites that are closely spaced, such as the anchor proteins at the cortex of fission yeast. Once dynein finds an anchor, it starts walking towards the minus end of the microtubule, thereby pulling on the microtubule and transporting the nucleus [26]. However, diffusion becomes less efficient at long-length scales, where directed transport is more efficient to reach a distant target. For example, dynein is transported by kinesins towards the plus end of the microtubule, where dynein loads its cargo to transport it to the minus end [36]. Thus, irrespective of whether dynein diffuses or is being transported along the microtubule, it switches to minus-end-directed movement upon reaching the target, in order to perform its biological function.

## Outlook

The concepts discussed in the present paper may apply to other motor proteins. We speculate that other motors also bind from the cytoplasm to the cytoskeleton fibres as the first step in their search for targets, in the case when they transport large cargos such as the nucleus in yeasts. For small cargos, future work will show whether motors bind first to the cytoskeleton or to the cargo.

When the motors are bound only to one binding partner, either cytoskeleton or cargo, they do not generate force. Because they cannot perform their biological function in this case, they may be inactive, as was shown for dyneins in fission yeast. When the motors subsequently find targets such as cortical anchors, organelles or other cytoskeleton-associated proteins, the motors may undergo activation of their intrinsic motility, which is required for their role in the cell. A straightforward approach based on the observation of single motors in a living cell and of key steps in their kinetics will help to uncover the behaviour of different motor proteins and thus to understand their function.
